# Prevalence, Etiology, and Associated Symptoms of Vaginal Discharge During Pregnancy in Women Seen in a Tertiary Care Hospital in Bihar

**DOI:** 10.7759/cureus.12700

**Published:** 2021-01-14

**Authors:** Dipali Prasad, Sadia Parween, Kanchan Kumari, Neelima Singh

**Affiliations:** 1 Obstetrics and Gynecology, Indira Gandhi Institue of Medical Sciences, Patna, IND; 2 Obstetrics and Gynecology, Indira Gandhi Institute of Medical Sciences, Patna, IND; 3 Microbiology Department, Indira Gandhi Institute of Medical Sciences, Patna, IND

**Keywords:** abnormal vaginal discharge, pregnancy, organism isolated, symptoms

## Abstract

Introduction

Vaginal discharge is the most frequent complaint during pregnancy, leading to numerous complications in both the mother and fetus.

Aim

The goal of this study was to determine the prevalence of vaginal discharge, investigate its common infectious causes and associated symptoms during pregnancy.

Methods

This hospital-based cross-sectional study performed over one year evaluated 200 expectant mothers with vaginal discharge at any trimester in the Department of Obstetrics and Gynecology, in cooperation with the Microbiology section, of Indira Gandhi Institute of Medical Science, Patna.

Results

The mean age of the mothers was 26.84±5.51 years (range 19-42 years). Most of the patients (47.5%) were in the age group of 26-35 years, belonged to the lower socioeconomic class (67.5%), gravida 3 or more (43.5%), and presented in the third trimester. The prevalence of pathological discharge in pregnancy was 148/308 (48.05%). A positive culture was obtained in 105 (52.5%), and negative culture was obtained in 95 (47.5%). Vaginal candidiasis was diagnosed in most cases (37.5%), followed by aerobic vaginitis (15%), trichomoniasis (13.0%), and bacterial vaginosis (8.5%). The non-pathological discharge was diagnosed in 26.0%. Dysuria was the most common symptom (32.5%), followed by itching (27.5%) and urinary tract infection (UTI; 10.0%). The following variables were significantly associated (P<0.05) with discharge: age (in years), age group, gravida, culture, organism isolated on culture, UTI as a symptom, and diagnosis.

Conclusion

Expectant mothers presenting with vaginal discharge need to be evaluated to identify the etiology and allow timely treatment, which might be helpful in preventing complications.

## Introduction

Vaginal discharge is a common reason for gynecological consultations. The female genital tract has a complex microbial flora, and cervical and vaginal secretions differ in quality and quantity depending on several factors, including age, menstrual period, and use of oral contraceptives. Vaginal discharge may be normal or abnormal. Physiological vaginal discharge in pregnancy is colorless or white, non-irritating, and odorless with no sequelae.

In contrast, abnormal vaginal discharge may be yellow, green, brown, or red, with a foul smell, pruritus, or dysuria depending on the cause of infection. Studies in developed countries have shown that up to 90% of vaginal discharge cases result from sexually transmitted infections [1,2]. The most common infection in pregnancy in most of the studies was *Candida albicans* infection, followed by bacterial vaginosis and *Trichomonas vaginalis* infection. A study conducted by Ekanem et al. in Nigeria reported the same observations [3].

Vaginal discharge may be related to bacterial vaginosis, Candida species, or *T. vaginalis*, whereas cervical discharge is usually caused by infection with *Neisseria gonorrhoeae* and/or *Chlamydia trachomatis* [4].

Some studies reported that the number of microorganisms isolated from pregnant women was higher than that in non-pregnant women. In most studies, the higher incidence rate of *C. albicans* in pregnant women than in non-pregnant women was attributed to increased estrogen content and glycosuria in the acidic vagina due to the rich glycogen content of the vaginal mucosa [5].

Infective vaginal discharge in pregnant women poses a great risk of complications, including abortion, premature rupture of membranes, chorioamnionitis, prematurity, low birth weight, and postpartum endometritis [6]. Owing to increased sensitivity and poor specificity, the syndromic approach to the treatment of vaginal discharge can result in overtreatment. The integration of antenatal screening in the form of laboratory testing for vaginal discharge has also been recommended [7].

Aim and objective

This study aimed to evaluate the common infectious causes of vaginal discharge, including bacterial vaginosis, vulvovaginal candidiasis, vaginal trichomoniasis, group B Streptococcus infection, and infection with other pathogens, in pregnant women who presented at our hospital.

## Materials and methods

This hospital-based cross-sectional study was performed in the Department of Obstetrics and Gynecology, in collaboration with the Microbiology section, of Indira Gandhi Institute of Medical Science, Patna. The present study was a cross-sectional study conducted over one year, from January 2018 to December 2018, in apparently healthy, married, pregnant women in the age group of 15-40 years who visited the obstetrics clinic and labor ward of our tertiary care hospital. The sample size was 200.

Inclusion criteria

All pregnant women who presented with vaginal discharge during their first antenatal visit in any trimester were considered to have met the inclusion criteria.

Exclusion criteria

The following women were excluded from the study: (i) pregnant women who had used antibiotics or vaginal medication in the previous 14 days and (ii) pregnant women with normal mucoid discharge, leaking, or bleeding per vaginum.

All patients were recruited irrespective of gestational age in their first antenatal visit. After providing written informed consent, the women underwent counseling accompanied by an interview in the clinic to collect information on sociodemographic factors. This was closely followed by clinical examinations involving a speculum examination and vaginal swabs for laboratory evaluations.

High vaginal swabs

Four high vaginal swabs were collected from the posterior fornix of the vagina of each patient and placed in sterile normal saline for culture (bacterial and fungal). The next three swabs were used for Gram staining, potassium hydroxide (KOH) test, and saline wet mount preparation. The vaginal swabs were also used for vaginal pH measurement and Nugent scoring.

Direct examination

Wet mounts were prepared by mixing swab samples with sterile normal saline on clean glass slides. The slides were examined under a microscope for typical yeast cells forming hyphae or pseudohyphae, and for *T. vaginalis*. Gram staining was performed on high vaginal swab samples, which were examined under a microscope with a 100× objective lens under oil immersion for Gram-negative diplococcus and clue cells. Bacterial vaginosis was defined according to the Nugent score.

Identification of isolates

Trichomoniasis was diagnosed on the basis of the findings of the wet preparation, whereas candidiasis was determined through a visual analysis of Candida species in KOH preparations or Gram-stained vaginal smears. The presence of other pathogens was detected from the cultures of specimens.

Statistical analysis

Data were coded and listed using the MS Excel spreadsheet application. SPSS version 23 (IBM Corp., Armonk, NY) was used for data analysis. Descriptive statistics are presented in the form of means with standard deviations and medians with interquartile ranges for continuous variables, and as proportions and frequencies for categorical variables. Data are graphically presented whenever befitting data visualization, using histograms/box-and-whisker plots/column graphs for continuous data and pub charts/pie graphs for categorical data. Group comparisons for continuously distributed data were performed with a completely independent-sample “t” evaluation when comparing two classes. When data were discovered to become non-normally distributed, appropriate non-parametric evaluations using the Wilcoxon test were also used. A chi-square evaluation was used for group comparisons for categorical data. In an event that the estimated prevalence in the contingency tables was found to function as <5 for >25% of the cells, Fisher's exact test was used. Statistical significance was set at P<0.05.

Ethical considerations

The study protocols were approved by the institutional ethical committee (memo no. 1265/Acad, dated November 30, 2016).

## Results

Of the 430 patients who attended the antenatal care clinic during the one-year study period, 308 patients met the eligibility criteria (increased vaginal discharge (with or without symptoms) and underwent per-speculum examination. A total of 108 patients presented with physiological discharge, whereas 200 patients presented with abnormal discharge and further underwent laboratory assessments. The prevalence of pathological discharge in the included pregnant women was 48.05% (148/308) and that of non-pathological discharge was 16.8% (52/308).

**Figure 1:**
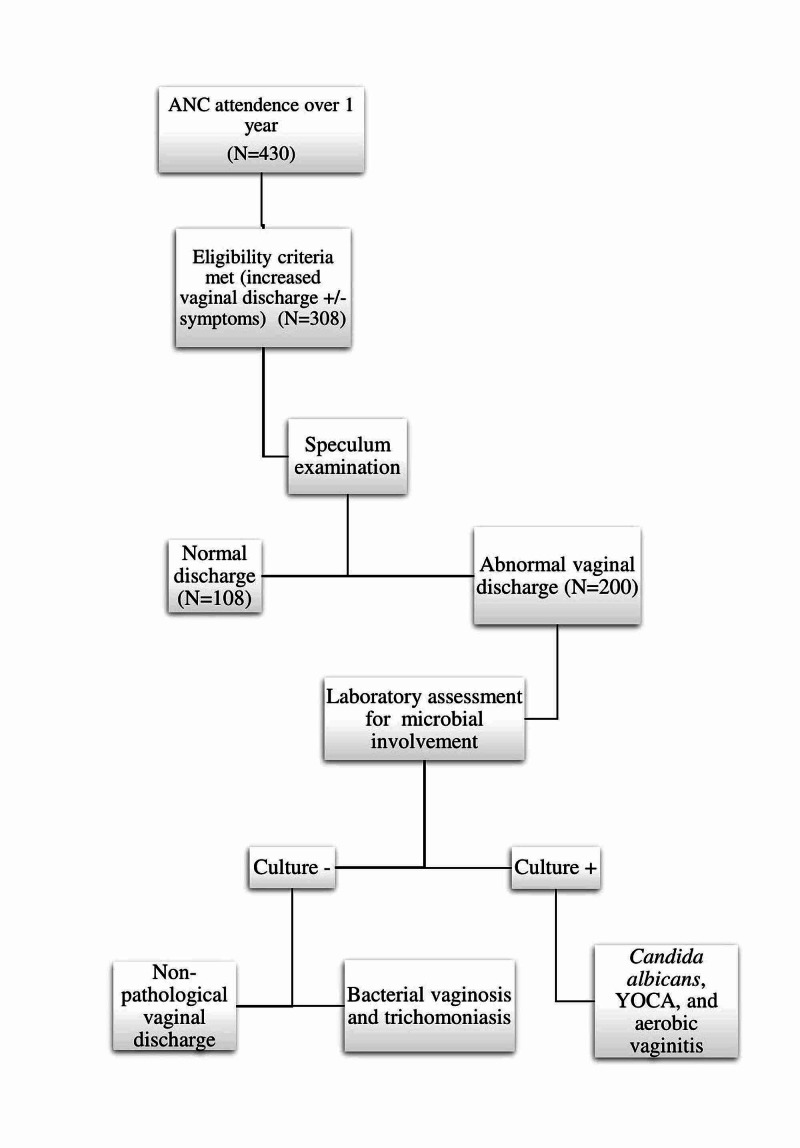
Flow of events. ANC: antenatal care, YOCA: yeast other than *C. albicans.*

The flowchart in Figure 1 describes the flow of events.

The mean age of the mothers was 26.84±5.51 years (Table 1). Of the patients, 91 (45.5%) were in the age group of 18-25 years, 95 (47.5%) were in the age group of 26-35 years, and 14 (7.0%) were in the age group of >35 years. Most of the patients (67.5%) belonged to the lower socioeconomic class. With respect to gravida, most (42.5%) were gravida 3 or more.

**Table 1:** Summary of all parameters (sociodemographic data, surgical, and medical history, symptoms, organism isolated, and diagnosis) PID: pelvic inflammatory disease, UTI: urinary tract infection, D&E: dilatation and evacuation, NUC: no use of contraception, IUCD: intra-uterine contraceptive devices, OCP: oral contraceptive devices, YOCA: yeast other than *C. albicans*.

Basic Details	Frequency (%)
Age group	
18-25 years	91 (45.5%)
26-35 years	95 (47.5%)
>35 years	14 (7.0%)
Socioeconomic status	
Upper	27 (13.5%)
Middle	38 (19.0%)
Lower	135 (67.5%)
Gravida	
G1	47 (23.5%)
G2	68 (34.0%)
G3	85 (42.5%)
Trimester	
First	29 (14.5%)
Second	64 (32.0%)
Third	107 (53.5%)
Medical/surgical history	
Not significant	52 (26.0%)
PID	67 (33.5%)
UTI	39 (19.5%)
D&E	32 (16.0%)
Diabetes	10 (5.0%)
Contraception history	
NUC	130 (65.0%)
IUCD	36 (18.0%)
OCP	34 (17.0%)
Discharge	
Thick curdy discharge	75 (37.5%)
Thin excessive discharge	52 (26.0%)
Purulent discharge	30 (15.0%)
Foul+frothy discharge	26 (13.0%)
Grayish-white+fishy discharge	17 (8.5%)
Symptoms: dysuria (present)	65 (32.5%)
Symptoms: itching (present)	55 (27.5%)
Symptoms: lower abdominal pain (present)	18 (9.0%)
Symptoms: redness (present)	50 (25.0%)
Symptoms: swelling (present)	35 (17.5%)
UTI (present)	20 (10.0%)
Culture (positive)	105 (52.5%)
Organism isolated on culture	
Negative	95 (47.5%)
C. albicans	42 (21.0%)
YOCA	33 (16.5%)
Escherichia coli	15 (7.5%)
Staphylococcus aureus	9 (4.5%)
Klebsiella pneumoniae	4 (2.0%)
Enterococcus species	2 (1.0%)
Diagnosis	
Vaginal candidiasis	75 (37.5%)
Non-pathological discharge	52 (26.0%)
Aerobic vaginitis	30 (15.0%)
Trichomoniasis	26 (13.0%)
Bacterial vaginosis	17 (8.5%)

Further, most of the patients were presented in the third trimester. Of the patients, 33.5% had a history of pelvic inflammatory disease, 19.5% had a history of urinary tract infection (UTI), 16.0% had a history of dilatation and evacuation, and 5.0% had a history of diabetes. Of the patients, 65.0% had no history of contraception, 18.0% had a history of use of an intrauterine contraceptive device, and 17.0% had a history of taking oral contraceptive pills. With respect to the characteristics of discharge, 37.5% of the patients had thick curdy discharge, 26.0% had thin excessive discharge, 15.0% had purulent discharge, 13.0% had foul+frothy discharge, and 8.5% had a grayish-white+fishy discharge.

Of the patients, 52.5% had a positive culture and 47.5% had a negative culture. Concerning organisms isolated on culture, 21.0% of the patients had *C. albicans*, 16.5% had yeasts other than *C. albicans* (YOCA), 7.5% had *E. coli*, 4.5% had *S. aureus*, 2.0% had *K. pneumoniae*, and 1.0% had Enterococcus species. The most common diagnosis was vaginal candidiasis (37.5%), followed by non-pathological discharge (26.0%), aerobic vaginitis (15.0%), trichomoniasis (13.0%), and bacterial vaginosis (8.5%). Dysuria was the most common symptom (32.5%), followed by itching (27.5%), redness (25.0%), swelling (17.5%), UTI (10.0%), and lower abdominal pain (9.0%).

**Figure 2:**
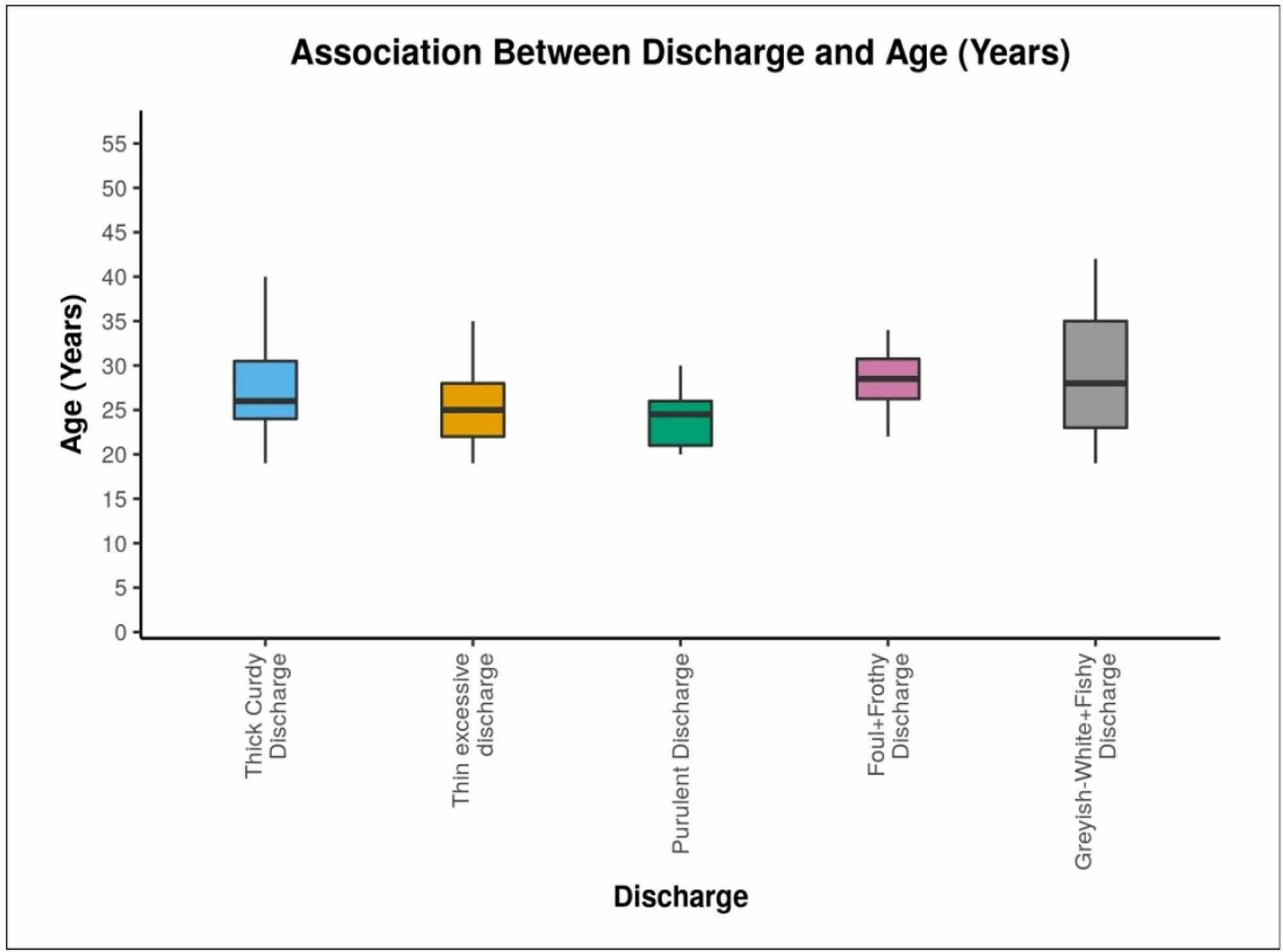
Box-and-whisker plot showing the association between discharge and age

Box-and-whisker plot in Figure 2 shows that patients with thick curdy discharge were in the age range of from 19 to 40 years (median 26 years), those with thin excessive discharge were aged 19-35 years (median 25 years), those with purulent discharge were aged 20-30 years (median 25 years), those with foul+frothy discharge were aged 21-34 years (median 29 years), and those with grayish-white frothy discharge were in the age range of 19-42 years (median 28 years). Overall, the mean (standard deviation) age was 26.84 (5.51) years, the median age was 26 years, and the age range was 19-42 years.

Table 2 shows that among primigravida women, the most common discharge was thin excessive discharge 15 (28.8%) and foul+frothy discharge was the least common discharge (7.7%). The thick curdy discharge was the most common 24 (32.0%) among gravida 2 women and 37 (49.3%) among gravida 3 women. The overall highest incidence of abnormal discharge (42.5%) was observed in those gravidae 3 or more. A significant difference was observed among the various groups in terms of the distribution of gravida (χ^2^=22.498, p=0.004).

**Table 2:** Comparison of five subgroups of discharge in terms of gravida (N=200)

Gravida	Discharge						Chi-square test	
	Thick curdy discharge	Thin excessive discharge	Purulent discharge	Foul+frothy discharge	Grayish-white+fishy discharge	Total	χ^2^	P-value
G1	14 (18.7%)	15 (28.8%)	13 (43.3%)	2 (7.7%)	3 (17.6%)	47 (23.5%)	22.498	0.004
G2	24 (32.0%)	22 (42.3%)	11 (36.7%)	7 (26.9%)	4 (23.5%)	68 (34.0%)		
G3 or more	37 (49.3%)	15 (28.8%)	6 (20.0%)	17 (65.4%)	10 (58.8%)	85 (42.5%)		
Total	75 (100.0%)	52 (100.0%)	30 (100.0%)	26 (100.0%)	17 (100.0%)	200 (100.0%)		

Table 3 shows that patients with vaginal discharge mostly presented with dysuria (32.5%), followed by itching (27.5%), redness (25.0%), swelling (17.5%), UTI (10%), and lower abdominal pain (9.0%).

**Table 3:** Comparison of five subgroups of discharge in terms of symptoms presented (N=200) UTI: urinary tract infection.

Symptoms	Discharge					
	Thick curdy discharge	Thin excessive discharge	Purulent discharge	Foul+frothy discharge	Grayish-white+fishy discharge	Total
Dysuria	31 (41.3%)	18 (25.0%)	9 (30.0%)	8 (30.8%)	4 (23.5%)	65 (32.5%)
Itching	22 (29.3%)	12 (23.1%)	8 (26.7%)	6 (23.1%)	7 (41.2%)	55 (27.5%)
Lower abdominal pain	5 (6.7%)	5 (9.6%)	4 (13.3%)	3 (11.5%)	1 (5.9%)	18 (9.0%)
Redness	20 (26.7%)	15 (28.8%)	6 (20.0%)	5 (19.2%)	4 (23.5%)	50 (25.0%)
Swelling	13 (17.3%)	10 (19.2%)	4 (13.3%)	5 (19.2%)	3 (17.6%)	35 (17.5%)
UTI	3 (4%)	5 (9.6%)	12 (40%)	0 (0.0%)	0 (0.0%)	20 (10%

Table 4 shows that positive culture was most frequently found in patients with thick curdy discharge, followed by those with purulent discharge. A negative culture was most frequently found in patients with thin excessive discharge, followed by those with foul+frothy discharge and those with grayish-white+fishy discharge.

**Table 4:** Association between discharge and culture (N=200)

Culture	Discharge						Chi-squared test	
	Thick curdy discharge	Thin excessive discharge	Purulent discharge	Foul+Frothy discharge	Grayish-white+fishy discharge	Total	χ2	P-value
Positive	75 (100.0%)	0 (0.0%)	30 (100.0%)	0 (0.0%)	0 (0.0%)	105 (52.5%)	200.000	<0.001
Negative	0 (0.0%)	52 (100.0%)	0 (0.0%)	26 (100.0%)	17 (100.0%)	95 (47.5%)		
Total	75 (100.0%)	52 (100.0%)	30 (100.0%)	26 (100.0%)	17 (100.0%)	200 (100.0%)		

Table 5 shows that *C. albicans* and YOCA were the most common organisms isolated from thick curdy discharge. *E. coli*, *S. aureus*, *K. pneumoniae*, and Enterococcus species were isolated from purulent discharge. A chi-square test was used to analyze the association between discharge and organism isolated on culture. A significant difference was observed among the various groups in terms of the distribution of organisms isolated on culture (χ^2^=400.000, P<0.001).

**Table 5:** Association between discharge and organism isolated on culture (N=200) YOCA: yeast other than *C. albicans*.

Organism isolated on culture	Discharge						Chi-square test	
	Thick curdy discharge	Thin excessive discharge	Purulent discharge	Foul+frothy discharge	Grayish-white+fishy discharge	Total	χ2	P-value
Negative	0 (0.0%)	52 (100.0%)	0 (0.0%)	26 (100.0%)	17 (100.0%)	95 (47.5%)	400.000	<0.001
C. albicans	42 (56.0%)	0 (0.0%)	0 (0.0%)	0 (0.0%)	0 (0.0%)	42 (21.0%)		
YOCA	33 (44.0%)	0 (0.0%)	0 (0.0%)	0 (0.0%)	0 (0.0%)	33 (16.5%)		
E. coli	0 (0.0%)	0 (0.0%)	15 (50.0%)	0 (0.0%)	0 (0.0%)	15 (7.5%)		
S. aureus	0 (0.0%)	0 (0.0%)	9 (30.0%)	0 (0.0%)	0 (0.0%)	9 (4.5%)		
K. pneumoniae	0 (0.0%)	0 (0.0%)	4 (13.3%)	0 (0.0%)	0 (0.0%)	4 (2.0%)		
Enterococcus species	0 (0.0%)	0 (0.0%)	2 (6.7%)	0 (0.0%)	0 (0.0%)	2 (1.0%)		
Total	75 (100.0%)	52 (100.0%)	30 (100.0%)	26 (100.0%)	17 (100.0%)	200 (100.0%)		

Table 6 shows that thick curdy discharge showed the largest proportion of all diagnoses (vaginal candidiasis, 37.5%), followed by thin excessive discharge (non-pathological discharge, 26.0%), purulent discharge (aerobic vaginitis, 15.0%), foul+frothy discharge (trichomoniasis, 13%), and grayish-white+fishy discharge (bacterial vaginosis, 8.5%). A chi-square test was used to analyze the association between discharge and diagnosis. A significant difference was observed among the various groups in terms of the distribution of diagnosis (χ^2^=800.000, P<0.001).

**Table 6:** Association between discharge and diagnosis (N=200)

Diagnosis	Discharge						Chi-square test	
	Thick curdy discharge	Thin excessive discharge	Purulent discharge	Foul+frothy discharge	Grayish-white+fishy discharge	Total	χ^2^	P-value
Vaginal candidiasis	75 (100.0%)	0 (0.0%)	0 (0.0%)	0 (0.0%)	0 (0.0%)	75 (37.5%)	800.000	<0.001
Non-pathological discharge	0 (0.0%)	52 (100.0%)	0 (0.0%)	0 (0.0%)	0 (0.0%)	52 (26.0%)		
Aerobic vaginitis	0 (0.0%)	0 (0.0%)	30 (100.0%)	0 (0.0%)	0 (0.0%)	30 (15.0%)		
Trichomoniasis	0 (0.0%)	0 (0.0%)	0 (0.0%)	26 (100.0%)	0 (0.0%)	26 (13.0%)		
Bacterial vaginosis	0 (0.0%)	0 (0.0%)	0 (0.0%)	0 (0.0%)	17 (100.0%)	17 (8.5%)		
Total	75 (100.0%)	52 (100.0%)	30 (100.0%)	26 (100.0%)	17 (100.0%)	200 (100.0%)		

## Discussion

Vaginal discharge is a common gynecological problem in women of childbearing age, affecting approximately one-third of all women and one-half of expectant mothers. The prevalence of pathological vaginal discharge was 48.05% in the present study, which was slightly more than the prevalence of 43% reported by Da Fonseca et al. in Brazil [8].

The above finding is not in line with that of a similar analysis done in Saudi Arabia that reported an increased incidence of 72.2% [9]. These greater prevalence rates were probably a result of inadequate health-seeking behavior combined with an inadequate understanding of perineal hygiene.

The following variables were significantly associated (P<0.05) with discharge: age (years), age group, gravida, culture, organism isolated on culture, UTI as a symptom, and diagnosis.

Of a total of 200 pregnant women with discharge on clinical evaluation who were recruited for the analysis, 52.5% tested culture-positive and 47.5% tested culture-negative in laboratory evaluations. Candidiasis (37.5%) was the most commonly diagnosed infection, followed by aerobic vaginitis (15.0%), including infections with *E. coli* (7.5%), *S. aureus* (4.5%), Klebsiella (2.0%), and Enterococcus species (1.0%). *T. vaginalis* (13.0%) and bacterial vaginosis (8.5%) were found on wet mount preparations of vaginal swabs, not by culture.

In this study, candidiasis was the most common cause of the pathological vaginal discharge. This was consistent with a cross-sectional study performed at a hospital in Western India, which found that 183 (78.54%) pregnant women had vaginal discharge, and the most common clinical diagnosis was *C. albicans* [10]. However, the study conducted in Nigeria reported a decreased incidence of 20% [11]. The high incidence of vaginal candidiasis has been attributed to poor hygiene, limited diagnostic process, and lack of effective therapy.

In this study, the prevalence of bacterial vaginosis was 13.6%. This rather correlates with the study done in Antenatal care at Kampala International University Teaching Hospital which revealed a prevalence of 10.1% [12].

The variations in the incidence rates of bacterial vaginosis have been attributed to sociodemographic traits, sexual practices, reproductive health, and genital hygiene.

The most common symptom reported in our study was dysuria (32.5%), followed by itching (27.5%), redness (25%), swelling (17.5%), UTI (10%), and lower abdominal pain (9%), while the study conducted in Saudi Arabia [9] reported itching (49.2%) followed by redness (48.4), dysuria (36%), and swelling (4.5%). In the present study, UTI (P<0.001) was significantly associated with variable discharge.

## Conclusions

Early diagnosis, treatment, and prevention of infective vaginal discharge in pregnant mothers will mitigate complications. A considerable number of pregnant women harbor pathogenic organisms that require early detection and treatment. Therefore, we highly recommend health education for pregnant women with respect to preventive measures in order to help prevent the spread of infection.
